# Profiling Dizziness in Older Primary Care Patients: An Empirical Study

**DOI:** 10.1371/journal.pone.0016481

**Published:** 2011-01-31

**Authors:** Jacquelien Dros, Otto R. Maarsingh, Daniëlle A. W. M. van der Windt, Frans J. Oort, Gerben ter Riet, Sophia E. J. A. de Rooij, François G. Schellevis, Henriëtte E. van der Horst, Henk C. P. M. van Weert

**Affiliations:** 1 Department of Family Medicine, Academic Medical Center, University of Amsterdam, Amsterdam, The Netherlands; 2 Department of Family Medicine and EMGO Institute for Health and Care Research, VU University Medical Center, Amsterdam, The Netherlands; 3 Arthritis Research Campaign National Primary Care Centre, Keele University, Staffordshire, United Kingdom; 4 Department of Medical Psychology, Academic Medical Center, University of Amsterdam, Amsterdam, The Netherlands; 5 Section of Geriatric Medicine, Department of Internal Medicine, Academic Medical Center, University of Amsterdam, Amsterdam, The Netherlands; 6 NIVEL, the Netherlands Institute for Health Services Research, Utrecht, The Netherlands; Los Angeles Biomedical Research Institute, United States of America

## Abstract

**Background:**

The diagnostic approach to dizzy, older patients is not straightforward as many organ systems can be involved and evidence for diagnostic strategies is lacking. A first differentiation in diagnostic subtypes or profiles may guide the diagnostic process of dizziness and can serve as a classification system in future research. In the literature this has been done, but based on pathophysiological reasoning only.

**Objective:**

To establish a classification of diagnostic profiles of dizziness based on empirical data.

**Design:**

Cross-sectional study.

**Participants and Setting:**

417 consecutive patients of 65 years and older presenting with dizziness to 45 primary care physicians in the Netherlands from July 2006 to January 2008.

**Methods:**

We performed tests, including patient history, and physical and additional examination, previously selected by an international expert panel and based on an earlier systematic review. We used the results of these tests in a principal component analysis for exploration, data-reduction and finally differentiation into diagnostic dizziness profiles.

**Results:**

Demographic data and the results of the tests yielded 221 variables, of which 49 contributed to the classification of dizziness into six diagnostic profiles, that may be named as follows: “frailty”, “psychological”, “cardiovascular”, “presyncope”, “non-specific dizziness” and “ENT”. These explained 32% of the variance.

**Conclusions:**

Empirically identified components classify dizziness into six profiles. This classification takes into account the heterogeneity and multicausality of dizziness and may serve as starting point for research on diagnostic strategies and can be a first step in an evidence based diagnostic approach of dizzy older patients.

## Introduction

Dizziness is a common symptom, especially in older patients. The prevalence of dizziness in the community ranges from 2% in young adults to over 30% in older people. Annual consultation rates in primary care increase from 3% for patients aged 25 to 44 years, to 8% in patients over 65 years of age, and to 18% for the oldest elderly [1]–[6].

The diagnostic approach to dizziness is often difficult for clinicians: dizziness is self-reported by definition, refers to various abnormal sensations of body orientation in space, and may be caused by a wide range of benign and serious conditions that may or may not co-exist in one patient [7]–[12]. Primary care physicians (PCPs) have to deal with unselected patients and in about a quarter of primary care patients presenting with dizziness no diagnosis is established, hampering effective management. Despite the high prevalence and the diagnostic difficulties empirical research on diagnosing dizziness is scarce [13]. In 1972 Drachman and Hart proposed a classification in four subtypes: vertigo (mainly caused by ear, nose, and throat (ENT) and neurological conditions), disequilibrium (mainly caused by orthopedic, neurological and/or sensory problems), presyncope (mainly caused by cardiac or vasomotor conditions) and atypical dizziness (mainly caused by psychiatric problems). This classification is generally accepted and frequently used since, but was not based on empirical evidence [14]. Therefore guidelines on the diagnostic strategy are mainly based on consensus and expert opinion [15]–[19].

The objective of our study was to establish an empirical classification of diagnostic profiles of dizziness in older patients, using information readily obtainable in a primary care setting in order to establish a starting point for a more specific diagnostic approach.

## Methods

### Participants

Participants were recruited among consecutive patients seen by 45 PCPs in 24 Dutch primary care practices from July 2006 to January 2008. Patients of 65 years or older consulting their PCP for dizziness were invited to participate. Additionally, each month the electronic databases of all practices were searched retrospectively for dizzy patients the PCPs had failed to invite. These patients received, after approval of their PCP, a written invitation to participate in the study. Our definition of dizziness included patients describing a giddy or rotational sensation, a feeling of imbalance, lightheadedness, and a sensation of impending faint. The complaints had to be present at inclusion and dizziness had to be the main reason for consultation. We included patients irrespective of prior consultations concerning the same symptoms. Criteria for exclusion were the inability to speak Dutch or English, severe cognitive impairment, severe visual impairment (i.e. corrected visual acuity of less than 3/60 for the best eye), severe hearing impairment (i.e. verbal communication impossible), or wheelchair dependency.

The study was approved by the medical ethics committees of both involved academic medical centers (Medical Ethics Committee Academic Medical Center Amsterdam (MEC AMC) and Medical Ethics Committee VU Medical Center (METc VUmc)). All patients gave written informed consent.

### Diagnostic tests

All patients were assessed by one of the authors (JD or OM) or one of three well-trained research assistants with a medical degree using a predefined protocol. The creation of the protocol is described elsewhere in more detail [20]. Briefly, after an extensive literature review we identified 36 tests, feasible in primary care and used to diagnose dizziness [21]. We presented test characteristics when available and other relevant information (like setting, and patient characteristics) of these tests to16 international experts, representing dizziness-relevant medical specialties. In a 3-round Delphi procedure these experts selected 21 tests as potentially contributing to the diagnostic process in older patients presenting with dizziness to a PCP; the tests included elements of patient history (4 items), physical examination (11 items), and additional diagnostic tests like the Patient Health Questionnaire (PHQ), an electrocardiogram and audiometry (6 items). In addition to these tests we gathered information on demographic variables, performed the validated timed ‘up and go’-test to measure functional mobility [22], and used the Dutch validated version of the Dizziness Handicap Inventory (DHI) [23]; [24] to quantify self-perceived impact of dizziness on everyday life.

Data were entered real-time in a database.

### Dataset

The full diagnostic test battery, including demographic data, consisted of 221 variables. We organized the dataset by merging and recoding (multi)nominal and ordinal variables into dichotomous variables [e.g. drugs use was originally described in 20 variables to register the names of all types of drugs used. We recoded these into five dichotomous variables (cardiovascular drugs yes/no, antivertiginous drugs yes/no, fall risk increasing drugs (FRID) yes/no, urologic drugs increasing blood pressure yes/no). Another example is the onset of dizziness; this was originally described in five variables (categories) and became one dichotomous variable, with 0. less than six months, and 1. six months or more]. We left continuous variables continuous (e.g. age, total amount of drugs, timed ‘up and go’-test in seconds), and dichotomous variables dichotomous (e.g. gender) (see supporting information). This process reduced the number of variables to 91. Next, we discarded variables that scored positive in less than 5% or in more than 95% of the patients and in addition were not indicative of acute (serious) conditions. Four variables were dropped: “epilepsy in history”, “urologic drugs increasing blood pressure”, “alcohol problem based on the PHQ”, and “abnormal non-fasting blood glucose”. All were dropped because of a very low prevalence, respectively 1.4%, 4.3%, 2.4%, and 1.4%.

We imputed missing data using the iterative chained equations method (ICE) in STATA/SE 10.0 (StataCorp, College Station, TX, USA). Briefly, for each variable in turn missing values are filled in with random predicted values based on observed values. Then “filled-in” values in the first variable are removed, leaving the original missing values for this variable. These missing values are then imputed using regression imputation on all other variables (inclusive their “filled-in” values). This process is repeated for each variable with missing values until one ‘cycle’ is completed. We continued this process for 5 cycles [25]; [26]. In this way 0.2% of all values were imputed.

### Data analysis

We used principal component analysis (PCA). PCA is a technique that can be used to summarize a large number of variables by a small number of components, wherein associated variables form a distinct pattern or profile. We performed PCA in a two-stepped procedure to mimic the diagnostic approach in daily practice. In the first step we used PCA to explore the inter-relationships of the demographic data and information from history taking. Variables with high loadings (≥+.35 or ≤−.35) [27]; [28] in the first step were used in a second step together with results from physical and additional examination. Both steps were done in SPSS 16.0 (SPSS Inc, Chicago, USA) with oblimin rotation and Kaiser normalisation. The number of components to be retained was in both analyses based on inspection of the scree plot [29], amount of explained variance, and examination of the component loading values.

The aim was to obtain as few components as possible whilst each variable loaded with a high value (≥+.35 or ≤−.35) onto one component and low values (−.34 to +.34) [27]; [28] onto the other components. We also examined cross loading of variables, defined as high loadings (≥+.35 or ≤−.35) of a variable on more than one component [27]; [28].

After completion of the PCA we calculated so-called “profile-scores” by summing, for each component, all variables associated with that component. These profile scores were standardised for each component to a score ranging from 0 to 100.

Internal consistency was estimated by calculating the Cronbach's alpha for each profile. Spearman correlation coefficients were calculated between profile-scores. Descriptive statistics were used to examine the distribution of the profile-scores in our population. Finally, profile-scores were dichotomized at a value of 67% in order to identify patients scoring high on specific components. The Mann-Whitney *U* test was used to test statistical differences in the distribution of the dizziness profiles by gender and age. Values of *p*<0.05 were considered statistically significant.

## Results

Data were available from 417 older patients with dizziness (Table 1) [30]. Their age ranged from 65 to 95 years with a mean age of 78.5 (SD = 7.1), 74% were female, and 69% experienced dizziness for more than half a year. For the first analytical step we used 57 variables concerning demographic data and patient history. This analysis identified 6 components which explained 29.4% of the variance. A total of 38 variables with component loading values ≥+.35 or ≤−.35 were considered contributively and were retained for the second step (Table S1).

**Table 1:** Patient characteristics of 417 dizzy older patients in primary care.

	No. (%) of patients
Sex, female	307 (73.6)
Age in years, mean (range)	78.5 (65–95)
Living situation	
Alone	254 (60.9)
In residential home	66 (15.8)
Ethnic background	
Dutch native	342 (82.0)
Western immigrant	31 (7.4)
Non-western immigrant	44 (10.6)
Level of education	
Elementary school	119 (28.5)
High school	247 (59.2)
College/university	51 (12.2)
Medical history	
Cardiovascular disease	205 (49.2)
Hypertension	239 (57.3)
Diabetes	78 (18.7)
Neurologic disease	145 (34.8)
Psychiatric disease	142 (34.1)
Onset of dizziness	
<6 months	128 (30.7)
≥6 months	289 (69.3)

**Figure 1:**
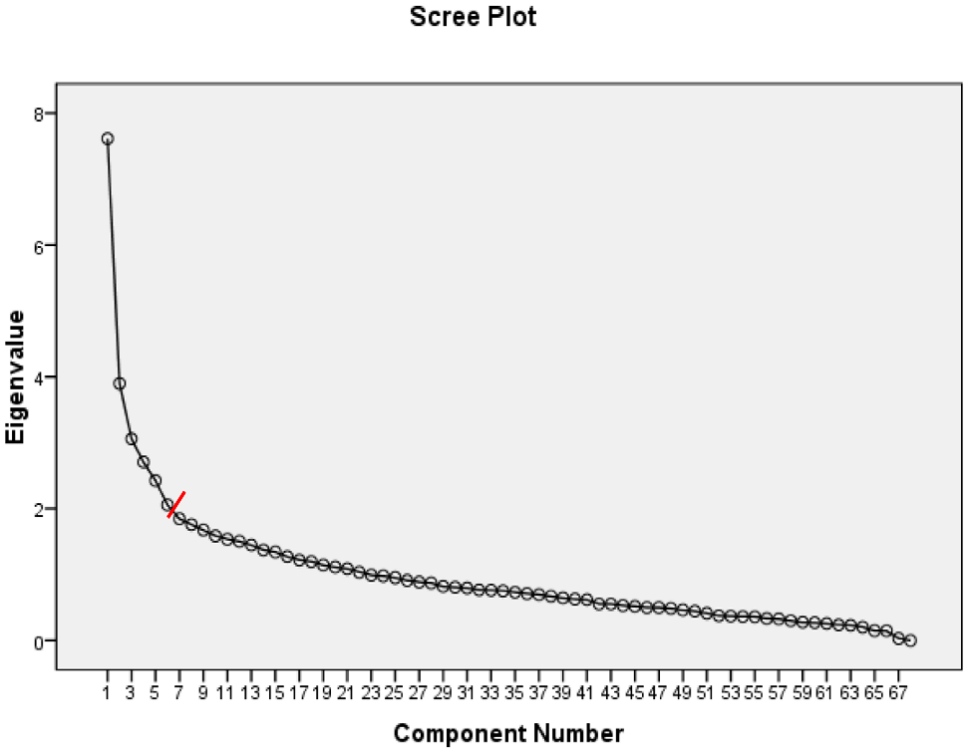
Scree plot of eigenvalues of 68 variables on demographic data, patient history, physical and additional examination, of 417 dizzy older primary care patients. Red line: cut off point.

In the second step we added 30 variables providing information on physical examination and additional diagnostic tests. Based on inspection of the scree plot (Figure 1) and component loading values PCA identified 6 components with 49 contributive variables, which explained 32.0% of the variance (Table S2). The 6 components were easily interpretable, were named as follows, and include the following variables:

1. “frailty”: (older) age, living in nursing home, living alone, using hearing and walking aid, impaired stability in rest, impaired walking (without walking aid), impaired mobility of knee joint(s), abnormal tandem gait, abnormal functional mobility (performance of the timed ‘up and go’ test ≥20 seconds), corrected visual acuity ≤0.5, impaired hearing (Fletcher index ≥35 dB).
2. “psychological”: female, history of anxiety and/or depressive disorder, use of psychotropic drugs; presence of somatoform disorder, major depressive disorder, and anxiety disorder (according to the Patient Health Questionnaire); high total score and high scores on emotional, functional and physical scales of DHI, indicating severe disability.
3. “cardiovascular”: history of cardiovascular disease, hypertension, history of arrhythmia, polypharmacy, use of cardiovascular drugs, use of fall risk increasing drugs (FRID).
4. “presyncope”: lightheadedness as subtype description of dizziness by patient, duration of dizziness >60 seconds, associated symptoms: tinnitus/decay in hearing; nausea; sweaty, pale or clammy; palpitations; chest pain; dyspnoea; trouble with walking; falling/almost falling; other symptoms.
5. “non-specific dizziness”: not frequently dizzy, absence of the following provoking circumstances: turning head, bending forward, looking up, getting up from lying or sitting position; negative Dix-Hallpike test; low total score and low score on physical scale of DHI.
6. “ENT”: ENT-disorders and/or ENT-surgery in history; use of antivertiginous drugs; using hearing aid; duration of dizziness 1 hour to days; nausea as associated symptom; impaired hearing (Fletcher index ≥35 dB).

“Frailty” accounted for 11.2% of the total variance, “psychological” for 5.7%, “cardiovascular” for 4.5%, “presyncope” for 4.0%, “non-specific dizziness” for 3.6%, and “ENT” for 3.0%.

Cross loading did not occur in the first step (Table S1). In the second step cross loading occurred for the use of hearing aids, nausea as associated symptom, impaired hearing, and for DHI-scores (total DHI score, and score on the physical scale of DHI) (Table S2).

Cronbach's alpha for each component was respectively .82 for “frailty”, .77 for “psychological”, .77 for “cardiovascular’, .69 for “presyncope”, .71 for “non-specific dizziness”, and .56 for “ENT”. This is satisfactory for most components (Cronbach's alpha ≥0.70), meaning that contributive variables in the different dizziness profiles are measuring the same underlying construct.

Table 2 shows the results of correlations between the six dizziness profiles, showing that the associations between dizziness profiles were almost all statistically significant (*p*<0.05), but not very strong for most components, confirming that the components represent different distinct presentations of dizziness. Higher correlations were found between the “psychological” and ”non-specific dizziness” profiles, and between the “psychological” and “ENT” profiles (correlation coefficients >0.50). Some higher correlation coefficients are inflated due to variables loading on more than one component (cross loading on “psychological” and “non-specific dizziness”, and “frailty” and “ENT”).

**Table 2:** Spearman correlation among dizziness profiles.

N = 417	Frailty	Psychological	Cardiovascular	Presyncope	Non-specific dizziness	ENT
Frailty	1					
Psychological	0.34**	1				
Cardiovascular	0.20**	0.13*	1			
Presyncope	0.14**	0.30**	0.12*	1		
Non-specific dizziness	0.13**	0.55**	0.08	0.20**	1	
ENT	0.46**	0.54**	0.12*	0.27**	0.41**	1

*Correlation is significant at the 0.05 level (2-tailed).

**Correlation is significant at the 0.01 level (2-tailed).

Spearman coefficients <0.3 indicate a weak correlation, 0.3–0.5 indicate a moderate correlation, and >0.5 indicate a strong correlation.

**Figure 2:**
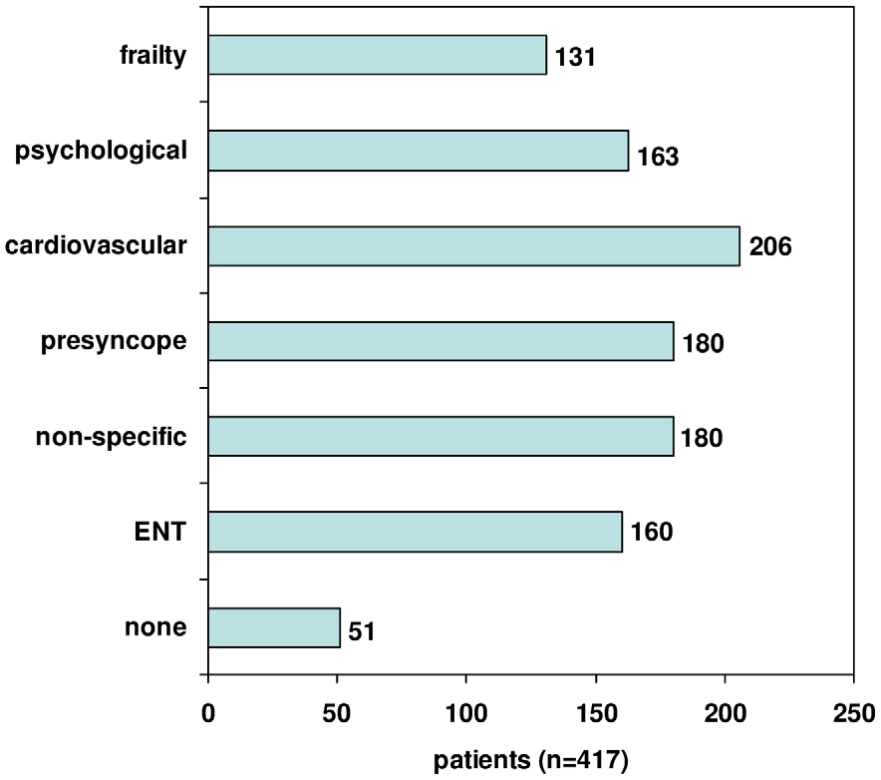
Distribution of patients over dizziness profiles. Based on profile scores in highest third. More than one profile per patient possible.

We classified the participating patients according to the identified profiles. For each of the dizziness profiles figure 2 shows the number of patients with a high score (≥67/100, the highest third). The results show that many patients scored high on multiple dizziness profiles. We were able to classify 366 (88%) of the patients, meaning that only 12% scored low on all six profiles, while 319 patients (76%) scored on more than one dizziness profile.

**Figure 3:**
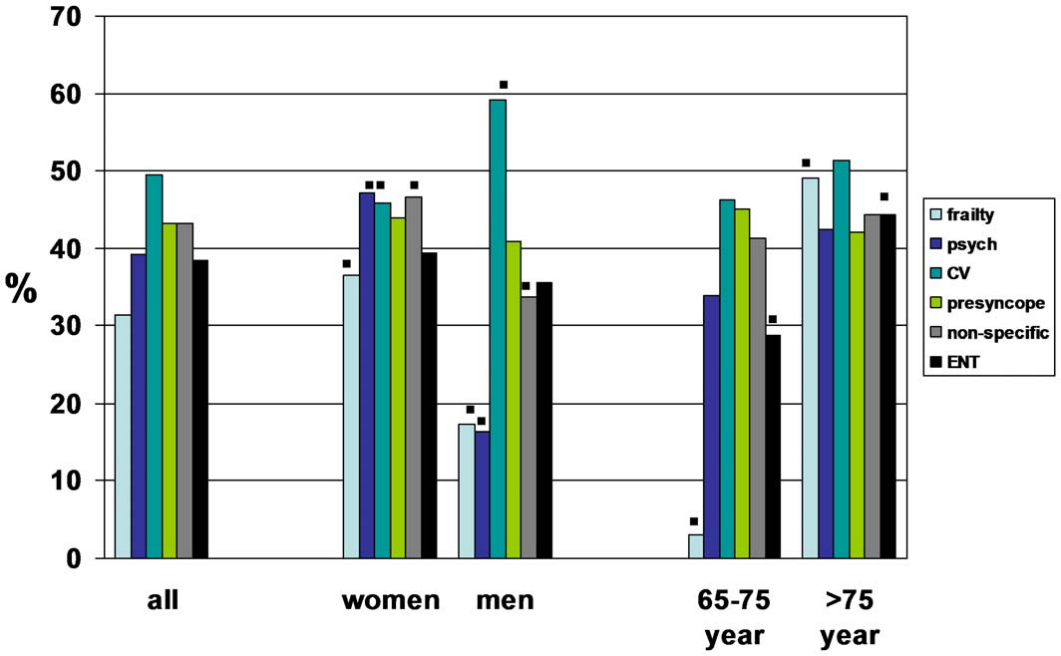
Distribution of dizziness profiles by gender and age. ▪significant at the 0.05 level (2-tailed).

Figure 3 shows the distribution of the dizziness profiles by gender and age. Women scored highest on the “psychological” profile, men scored highest on the “cardiovascular” profile. Both the younger old and the older old scored highest on the “cardiovascular” profile. While the older old scored also high on the “frailty” profile, the younger old scored low on several profiles, in particular “frailty”.

## Discussion

Based on findings from history, physical examination and diagnostic tests, that can be performed in a primary care setting, we found six dizziness profiles: “frailty”, “psychological”, “cardiovascular”, “presyncope”, “non-specific dizziness”, and “ENT”. The vast majority of patients (in this study 88%) could be classified in one of six profiles.

The components identified by the first PCA step, based on demographic data and patient history were quite similar to the components of the second step, in which we added physical examination and diagnostic tests. Additional information on physical examination and diagnostic tests seems to be redundant and only confirmed the diagnostic information provided by demographic data and patient history. This means that a thorough history taking seems to be sufficient for the initial classification of dizziness. This fact does not mean that additional tests should be abandoned, as these tests might be useful within a profile for diagnosing or ruling out a specific disease [21].

The existing pathophysiological classification of dizziness used four subtypes: vertigo, disequilibrium, presyncope, and atypical dizziness. This classification shows resemblance with our results: “ENT” combined with the absence of “non-specific dizziness” resemble vertigo. “Frailty”, although much broader, resembles disequilibrium, “cardiovascular” and “presyncope” together resemble presyncope, and “psychological”, although more specific, resembles atypical dizziness. The empirically established profiles thus provide a more in depth addition to the existing theoretical classification.

To our knowledge this is the first study that used empirical data to define profiles of dizziness in an unselected older primary care population presenting with dizziness [31]. We only used information that is readily available in primary care, as this information will be gathered as a first diagnostic step. We selected diagnostic tests based on the results of a Delphi procedure among an international expert panel. We used a broad definition of dizziness to reflect clinical practice as much as possible. Our dataset was quite complete, with only 0.2% data missing.

The explained variance of the identified profiles of 32% is only moderate. This percentage is to be expected within the broad, heterogeneous spectrum of patients with dizziness in our dataset [28].

We did find cross loading. We retained all cross loaders, however, as all these variables showed sufficiently high factor loadings and add relevant information to the profiles. The use of hearing aids and impaired hearing, for instance, can be expected in both “frailty” and “ENT”, as older patients often have sensory deficits, and patients with (a history of) ENT problems may both have impaired hearing and use hearing aids. This is similar for nausea, which can be an associated symptom in vertigo (associated with “ENT”) as well as in presyncope. Furthermore, high total scores on the self-perceived Dizziness Handicap Inventory are to be expected in the “psychological” dizziness profile and the opposite (negative cross loading) in the relatively healthy “non-specific dizziness” group.

We performed the study in an older primary care population suffering from dizziness for at least two weeks, often for a long time. Our results therefore may not be applicable to other populations, like younger patients with acute onset of dizziness. In acute dizziness, however, diagnostic problems are less extensive.

From our study emerged six dizziness profiles that might provide clinical guidance in the diagnostic approach of dizzy older primary care patients. We were able to classify 88% of the patients, mostly in more than one profile. The fact that most patients scored positive on more than one profile could be expected as dizziness in the elderly is often a multifactorial problem. These profiles, based on readily available information during a consultation, might however guide the diagnostic process, thereby limiting the number of diagnostic tests which are needed to reach a more precise diagnosis. Instead of performing a complete diagnostic work-up for dizziness, PCP's might be able to taper the diagnostic process, using these dizziness profiles.

Before implementing this classification of dizziness profiles, external validation in another older primary care patient population with dizziness is mandatory. An empirical classification of dizziness profiles might serve as starting point for further research on diagnostic strategies, diagnostic test accuracy or prediction models within a more homogeneous group of patients presenting with dizziness. Then, within these profiles, an attending doctor can explore diagnostic options with uncomplicated and inexpensive testing and look for treatable conditions, etiologic or contributory.

## Supporting Information

Table S1 **Principal component analysis of demographic data, and patient history (first step).** Principal component analysis with OBLIMIN rotation and Kaiser normalisation. All component loadings are rounded to two decimals. Component loadings of ≥+.350 or ≤−.350 are deemed contributive and highlighted in bold. Empty cells represent component loadings of −.004 to +.004. *Continuous variables, all other variables are binary. We performed principal component analysis (PCA) in a two-stepped procedure to mimic the diagnostic approach in daily practice. In the first step we used PCA to explore the inter-relationships of the demographic data and information from history taking (57 variables). This analysis identified 6 components which explained 29.4% of the variance. A total of 38 variables with component loading values ≥+.35 or ≤−.35 were considered contributively and were retained for the second step. The results of this first analytic step with PCA resemble the results of the second step which also included physical examination and additional testing, with the component “healthy” being the opposite of the component “frailty”, and the component “vestibular” the opposite of the component “non-specific dizziness” (table S1 and table S2).
(DOC)

Table S2 **Principal component analysis of contributing demographic data and patient history, and physical examination, and additional information (second step).** Principal component analysis with OBLIMIN rotation and Kaiser normalisation. All component loadings are rounded to two decimals. Component loadings of ≥+.350 or ≤−.350 are deemed contributive and highlighted in bold. Empty cells represent component loadings of −.004 to +.004. *Continuous variables, all other variables are binary.
(DOC)
