# Supplementary material for: Profiling Dizziness in Older Primary Care Patients: An Empirical Study
Source: PLoS One. 2011 Jan 31;6(1):e16481. doi: 10.1371/journal.pone.0016481 (PMC3031582; doi:10.1371/journal.pone.0016481)
Supplement: Table S1 — Principal component analysis of demographic data, and patient history (first step). Principal component analysis with OBLIMIN rotation and Kaiser normalisation. All component loadings are rounded to two decimals. Component loadings of ≥+.350 or ≤−.350 are deemed contributive and highlighted in bold. Empty cells represent component loadings of −.004 to +.004. *Continuous variables, all other variables are binary. We performed principal component analysis (PCA) in a two-stepped procedure to mimic the diagnostic approach in daily practice. In the first step we used PCA to explore the inter-relationships of the demographic data and information from history taking (57 variables). This analysis identified 6 components which explained 29.4% of the variance. A total of 38 variables with component loading values ≥+.35 or ≤−.35 were considered contributively and were retained for the second step. The results of this first analytic step with PCA resemble the results of the second step which also included physical examination and additional testing, with the component “healthy” being the opposite of the component “frailty”, and the component “vestibular” the opposite of the component “non-specific dizziness” (table S1 and table S2). (DOC) [file pone.0016481.s001.doc]

**Table S1. Principal component analysis of demographic data, and patient history.**

| **Principal components** | | | | | | |
| --- | --- | --- | --- | --- | --- | --- |
|  | Healthy | Psychological | Cardiovascular | Presyncope | Vestibular | ENT |
| **Demographic** | | | | | | |
| Age* | **-.68** | -.03 | .02 | -.07 | .09 | -.23 |
| Sex (f=1) | -.27 | **.55** | -.21 | -.09 | -.01 | .02 |
| Ethnicity | .01 | .07 | -.03 | .10 | -.30 | .15 |
| Living in residential home | **-.42** | .11 |  | .16 | -.04 | -.17 |
| Living alone | **-.49** | .31 | -.11 | -.03 | -.16 | -.03 |
| Education | .13 | -.26 | .01 | -.09 | .03 | .19 |
| **Patient history** | | | | | | |
| Cardiovascular disease | -.05 | -.25 | **.59** | .23 | .02 | -.18 |
| Hypertension | .08 | .18 | **.62** | -.03 | -.12 | .07 |
| Arrhythmia | .05 | -.20 | **.44** | .27 | .03 | -.28 |
| CVA + TIA | -.11 | .03 | .14 | .06 | .21 | -.12 |
| Diabetes | -.05 | -.07 | .32 | -.05 | -.01 | .09 |
| Orthopaedic disorder | -.31 | .04 | .13 | -.04 | .20 | .07 |
| ENT | -.04 | -.05 | .07 | -.15 | .22 | **.47** |
| Anxiety and/or depressive disorder | -.08 | **.49** | .01 | .06 | .05 | .06 |
| Thyreoid dysfunction |  | **.37** | -.02 | -.07 | .11 | -.02 |
| Cataract and/or macula degeneration | -.26 | .01 | .03 | -.01 | .01 | -.08 |
| Often unexplained complaints | .06 | .29 | .11 | .10 | .10 | .01 |
| *Drugs* | | | | | | |
| Total amount of drugs* | -.09 | .04 | **.74** | .08 | .08 | -.03 |
| Cardiovascular drugs |  | .08 | **.88** | -.07 | -.06 | .02 |
| Psychotropic drugs | -.14 | **.56** | .12 | -.09 | .05 | .08 |
| Antivertigo drugs | .07 | -.05 | .07 | -.04 | .07 | **.40** |
| Fall risk increasing drugs (FRID) | -.10 | .17 | **.81** | -.14 | -.05 | .18 |
| Smoking | .22 | .10 | -.02 |  | -.08 | -.13 |
| Alcoholintake | .20 | -.11 | -.04 | -.05 | .09 | -.05 |
| *Use of medical aids* | | | | | | |
| Hearing aid | **-.37** | .01 | .07 | -.08 | -.02 | .07 |
| Glasses | -.09 | -.19 |  | .14 | .09 | -.32 |
| Walking aid | **-.66** | .11 | .11 | .11 | .10 | -.22 |
| *Subtype description dizziness* | | | | | | |
| Lightheadedness | .07 | -.01 | -.01 | **.39** | .06 | -.04 |
| Spinning sensation | .13 | .06 | -.03 | .03 | .28 | **.40** |
| Unsteadiness | -.16 | -.04 | .02 | .22 | .19 | .18 |
| Not classifiable | .17 | .07 | -.03 | .26 | .17 | .02 |
| Onset of dizziness | -.04 | .13 | .03 | .16 | .16 | .18 |
| Frequency of dizziness | -.17 | .03 | -.01 |  | **.50** | -.12 |
| *Duration of dizziness* | | | | | | |
| <60 seconds | .18 | -.04 | .12 | **-.45** | .22 | -.34 |
| 1min-1hour |  | **.37** |  | .10 | -.16 | -.09 |
| 1hour-days | -.07 | -.05 | -.03 | .10 | -.24 | **.54** |
| combination of possibilities | -.16 | -.30 | -.13 | **.39** | .11 | .04 |
| *Provoking circumstances* | | | | | | |
| Standing still | -.08 | .17 | -.04 | .34 | .12 | -.18 |
| Exercise | .35 | .22 |  | .28 | .13 | -.13 |
| Turning head | .13 | .14 | -.11 | .01 | **.60** | .16 |
| Bending forward | .03 | .16 | -.06 | .10 | **.60** | .04 |
| Looking up | .05 | .07 | -.06 | .06 | **.61** | .01 |
| Strong emotions | .20 | **.45** | -.02 | .20 |  | -.01 |
| Getting up from lying or sitting position | -.10 | -.08 | .02 | .06 | **.53** | .05 |
| Other | -.03 | .06 | -.06 | .06 | -.19 | -.05 |
| *Associated symptoms* | | | | | | |
| Tinnitus/decay in hearing | .02 | .11 | .04 | .35 | -.08 | **.35** |
| Central neurological (e.g. diplobia, dysphagia) | .02 | .11 | .03 | .34 | .08 | .11 |
| Nausea | .09 | -.09 | .03 | .30 | .01 | **.52** |
| Sweaty, pale, or clammy | .24 | .03 |  | **.50** | -.07 | .18 |
| Palpitations | .13 | .17 | -.01 | **.44** | .03 | -.08 |
| Chest pain | .17 | .13 | .06 | **.53** | .01 | -.19 |
| Dyspnoea | .12 | .16 | .14 | **.56** | -.04 | -.17 |
| Fear | -.14 | .27 | -.02 | .34 | -.25 | .14 |
| Trouble with walking | -.34 | -.11 | -.01 | **.59** | .05 | .05 |
| Falling/almost falling | -.33 | -.14 |  | **.58** | .06 | .03 |
| Other symptoms | -.10 | -.08 | .05 | **.39** | -.15 |  |
| Avoidance of activities because of dizziness | -.29 | .05 | -.01 | .25 | .24 | .15 |
