# Supplementary material for: Profiling Dizziness in Older Primary Care Patients: An Empirical Study
Source: PLoS One. 2011 Jan 31;6(1):e16481. doi: 10.1371/journal.pone.0016481 (PMC3031582; doi:10.1371/journal.pone.0016481)
Supplement: Table S2 — Principal component analysis of contributing demographic data and patient history, and physical examination, and additional information (second step). Principal component analysis with OBLIMIN rotation and Kaiser normalisation. All component loadings are rounded to two decimals. Component loadings of ≥+.350 or ≤−.350 are deemed contributive and highlighted in bold. Empty cells represent component loadings of −.004 to +.004. *Continuous variables, all other variables are binary. (DOC) [file pone.0016481.s002.doc]

**Table S2**. Principal component analysis of demographic data, patient history, physical examination, and additional information.

|  | **Principal components** | | | | | |
| --- | --- | --- | --- | --- | --- | --- |
|  | Frailty | Psychological | Cardio-vascular | Presyncope | Non-specific dizziness | ENT |
| **Demographic** | | | | | | |
| Age* | **.77** | -.08 | -.02 | -.14 | .08 | .10 |
| Sex (f=1) | .14 | **.47** | -.22 | -.05 | .01 | -.18 |
| Living in residential home | **.45** | .06 | -.04 | .16 | .09 | -.06 |
| Living alone | **.37** | .25 | -.14 | .01 | .21 | -.04 |
| **Patient history** | | | | | | |
| Cardiovascular disease | .05 | -.19 | **.63** | .13 | -.07 | -.04 |
| Hypertension | -.11 | .14 | **.60** | -.04 | .12 | -.04 |
| Arrhythmia | -.01 | -.18 | **.47** | .18 | -.09 | -.06 |
| ENT | -.03 | -.01 | .02 | -.07 | -.19 | **.50** |
| Anxiety and/or depressive disorder | -.03 | **.46** | .03 | .14 | .03 | -.14 |
| Thyreoid dysfunction |  | .25 | -.02 | -.04 | -.09 | -.10 |
| *Drugs* | | | | | | |
| Total amount of drugs* | .04 | .13 | **.72** | .04 | -.01 | -.02 |
| Cardiovascular drugs | -.07 | .08 | **.88** | -.09 | .09 | -.01 |
| Psychotropic drugs | .06 | **.55** | .13 | -.01 | .12 | .01 |
| Antivertigo drugs | -.09 | .05 | .03 | -.02 | -.14 | **.38** |
| Fall risk increasing drugs (FRID) | -.05 | .18 | **.80** | -.10 | .14 | .11 |
| *Use of medical aids* | | | | | | |
| Hearing aid | **.36** | -.06 | .03 | -.10 | .16 | .38 |
| Walking aid | **.72** | .08 | .08 | .05 | -.03 | -.08 |
| *Subtype description dizziness* | | | | | | |
| Lightheadedness | -.07 | -.08 | .02 | **.43** | -.03 | -.01 |
| Spinning sensation | -.14 | -.02 | -.07 | .09 | -.28 | .21 |
| Frequency of dizziness | .18 | .17 | .04 | -.15 | **-.48** | .01 |
| *Duration of dizziness* | | | | | | |
| <60 seconds | -.04 | -.15 | .15 | **-.56** | -.12 | -.16 |
| 1min-1hour | -.01 | .23 | .01 | .17 | .20 | -.20 |
| 1hour-days | -.09 | .05 | -.08 | .27 | .22 | **.39** |
| combination of possibilities | .15 | -.11 | -.12 | .29 | -.26 | .06 |
| *Provoking circumstances* | | | | | | |
| Turning head | -.13 | .11 | -.08 |  | **-.53** | .05 |
| Bending forward | .05 | .11 | -.05 | .02 | **-.57** | .04 |
| Looking up | .04 | .01 | -.04 | -.02 | **-.57** | -.02 |
| Strong emotions | -.14 | .32 | -.01 | .25 | .03 | -.06 |
| Getting up from lying or sitting position | .11 | -.11 | .05 | .01 | **-.48** | .12 |
| Tinnitus/decay in hearing | -.02 | .07 | -.04 | **.42** | .07 | .25 |
| *Associated symptoms* | | | | | | |
| Nausea | -.23 | .01 | .03 | **.38** | -.07 | **.40** |
| Sweaty, pale, or clammy | -.24 | .05 | .02 | **.53** | -.02 | .04 |
| Palpitations | -.02 | .13 |  | **.41** | -.06 | -.11 |
| Chest pain | .04 | .09 | .04 | **.48** | -.03 | -.21 |
| Dyspnoea | .07 | .16 | .11 | **.51** | .01 | -.21 |
| Trouble with walking | .29 | .02 | .01 | **.50** | -.14 | .01 |
| Falling/almost falling | .27 |  | .02 | **.49** | -.14 | .01 |
| Other symptoms | .07 |  | .04 | **.38** | .11 | -.01 |
| **Physical examination** | | | | | | |
| *Cardiovascular system* | | | | | | |
| Pulse measurement | -.01 | -.23 | .18 | .18 |  | .02 |
| Irregular pulse | .17 | -.18 | .13 | .06 | -.05 | -.03 |
| Auscultation of the heart | .16 | .03 | .16 | -.08 | -.04 | -.16 |
| Orthostatic hypotension | -.03 | .05 | .18 | .02 | .07 | .14 |
| *Locomotor system* | | | | | | |
| Stability in rest | **.62** | .16 | .07 | -.10 | -.12 |  |
| Walking (without walking aid) | **.52** | .06 | .15 | .01 | -.21 | -.14 |
| Mobility hip joints | .23 | -.20 | .03 | .24 | -.21 | -.11 |
| Mobility knee joints | **.36** | .05 | .08 | .06 | -.13 | -.15 |
| Mobility ankle joints | .32 | -.05 | .04 | .26 | .04 | -.05 |
| Tandem gait, feet outside the line* | **.75** | .03 | -.06 | .07 | -.10 | -.09 |
| Tandem gait, in sec* | **.68** | .12 |  | .22 | -.15 | -.14 |
| TUG, in sec* | **.67** | .19 | .02 | .24 | -.09 | -.13 |
| *Neurological system* | | | | | | |
| Achilles tendon reflex | .14 | .01 | .15 | -.08 | .12 | .01 |
| Plantar reflex | .04 | -.03 | -.01 | .13 | .16 | .13 |
| Peripheral neuropathy with monofilament test | .26 | -.06 | .09 | -.09 | -.03 | -.06 |
| *Vestibular/auditory system* | | | | | | |
| Otoscopy | -.03 | -.01 | .09 | -.12 | -.03 | .25 |
| Dix-Hallpike maneuver | -.13 | -.04 | -.01 | .05 | **-.46** | -.14 |
| *Other* | | | | | | |
| Visual acuity* | **.43** | .09 | -.03 | -.02 | .07 | .11 |
| **Additional examination** | | | | | | |
| Anaemia based on haemoglobin | .08 | -.02 | .15 | -.01 | -.05 | .06 |
| Audiometry right | **.55** | -.11 | -.03 | -.11 | .26 | **.39** |
| Audiometry left | **.51** | -.09 | -.03 | -.03 | .25 | **.42** |
| Residual ischaemic damage on ECG | .02 | -.12 | .18 | .25 | -.04 | -.08 |
| Arrythmia on ECG and/or cardiac event recording | .08 | -.28 | .17 | .10 | -.19 | .10 |
| *Psychiatric with Patient Health Questionnaire* | | | | | | |
| Somatoform disorder | .05 | **.46** | .16 | .02 | -.16 | .19 |
| Major depressive disorder | .15 | **.43** | .12 | -.02 | -.08 | .18 |
| Anxiety disorder | .06 | **.41** | .09 | .13 | -.07 |  |
| *Dizziness Handicap Inventory (DHI)* | | | | | | |
| DHI-score* | .26 | **.60** | .09 | .16 | **-.40** | .34 |
| Score DHI functional scale* | .28 | **.52** | .14 | .11 | -.30 | .21 |
| Score DHI emotional scale* | .19 | **.60** | .04 | .16 | -.17 | .32 |
| Score DHI physical scale* | -.03 | **.41** | .06 | .08 | **-.57** | **.35** |
